# Supplementary material for: PLGA based formulations with poly(2-oxazoline)s for controlled dexamethasone release from thin extrudates
Source: Int J Pharm X. 2026 Apr 1;11:100532. doi: 10.1016/j.ijpx.2026.100532 (PMC13091157; doi:10.1016/j.ijpx.2026.100532)
Supplement: Supplementary file 1 — Supplementary material [file mmc1.docx]

International Journal of Pharmaceutics X

PLGA based formulations with poly(2-oxazoline)s for controlled dexamethasone release from thin extrudates

Philipp S. Hilgeroth^a,b^, Eric Lehner^c^, Juliana Martins-Schalinski^b,d^, Karsten Mäder^e^, Wolfgang H. Binder^a,b^*

^a^Institute of Chemistry, Martin Luther University Halle-Wittenberg, Von-Danckelmann-Platz 4, 06120 Halle (Saale), Germany

^b^Design of 3D-Printable Polymers Based on Regional Resources, Just Transition Center, Martin Luther University Halle-Wittenberg, 06099 Halle, Germany

^c^Department of Otorhinolaryngology-Head and Neck Surgery, Martin Luther University Halle-Wittenberg, Ernst-Grube-Straße 40, 06120 Halle (Saale), Germany

^d^Institute of Physics, Martin Luther University Halle-Wittenberg, Heinrich-Damerow-Str. 4, 06120 Halle (Saale), Germany

^e^Institute of Pharmacy, Martin Luther University Halle-Wittenberg, Wolfgang-Langenbeck-Straße 4, 06120 Halle (Saale), Germany

Abstract:

PLGA is a landmark in polymer-based drug delivery, due to its biocompatibility, biodegradability and great mechanical strength. However, its high mechanical resistance tends to be unfavorable for implantation, as it creates sharp edges upon rupture, which can cause damage to the patient. Adding polyethylene glycol (PEG) as plasticizer reduces stiffness in PLGA blends, however with significant drawbacks due to deformation upon implantation. This motivates our evaluation of poly(2-alkyl-oxazoline)s (POx) as an alternative plasticizer for PLGA, probing its mechanical properties and the release behavior of dexamethasone (Dex) from POx/PLGA-based blends. Poly(2-oxazoline)s with defined chain length bearing different alkyl side chains were synthesized using a microwave assisted living polymerization, and characterized using gel permeation chromatography (GPC) and matrix assisted laser desorption ionization time of flight (MALDI-ToF) mass spectrometry. To achieve a homogeneous distribution of the POx inside the PLGA-polymer blend, polymer powders were cryo-milled and subsequently extruded, creating thin filaments as possible candidates for *in vivo* implantation. In addition to the *in vitro* drug release, the mechanical properties of the filaments were studied using melt rheology and texture analysis. Phase separation and micro phase behavior was observed using differential scanning calorimetry (DSC) and wide angle x-ray scattering (WAXS). A favorable plasticizing effect of the poly(2-alkyl-oxazoline)s in the PLGA blends was observed, reducing the lag time and increasing the drug release kinetics. Significant improvements in their mechanical properties prospects their use in future intracochlear implants, especially for the PEtOx containing formulations.

Keywords: Biodegradable polymer, Poly(2-alkyl-oxazolin)e, PLGA, Dexamethasone, Controlled release

Graphical Abstract


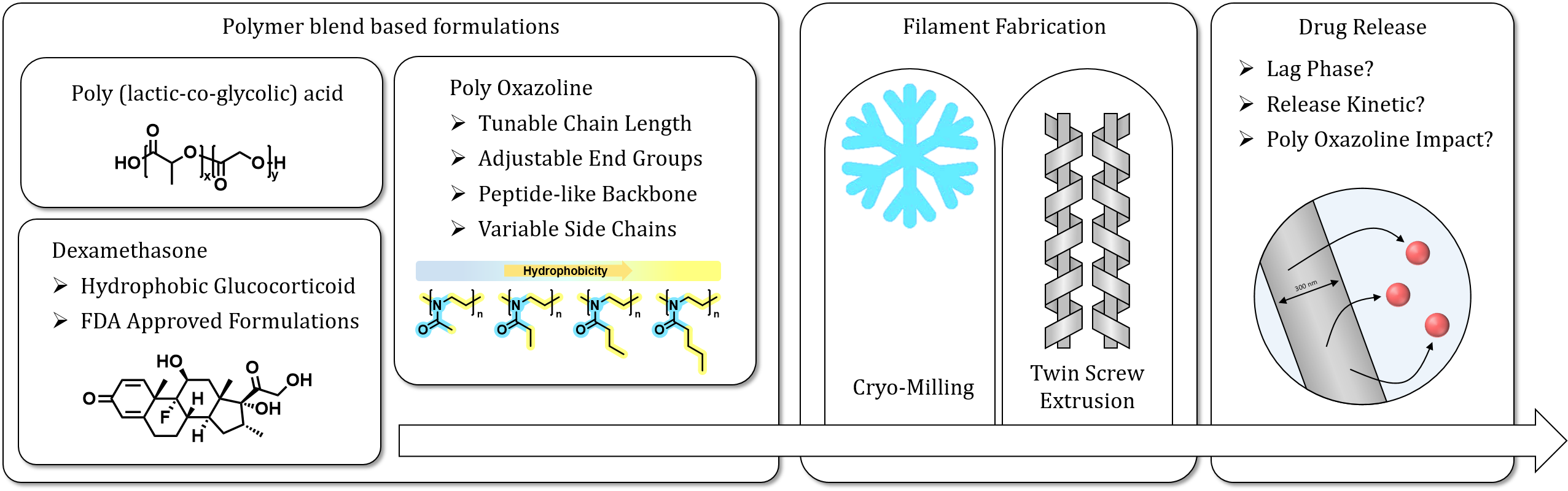


1. Introduction

Poly(lactic-co-glycolic) acid (PLGA) is a synthetic co-polymer composed of lactic acid (LA) and glycolic acid (GA), approved by the U.S. Food and Drug Administration (FDA)^1^ and European Medicines Agency (EMA)^2^ for medical use as a hydrophobic and biodegradable co-polymer. Through its versatile chemical nature, the LA:GA ratio can be adjusted to suite different applications, especially drug delivery, where biocompatible, biodegradable polymers are sought-after. A high GA content results in a more hydrophilic co-polymer that facilitates a faster degradation, whereas a high LA content makes the polymer more hydrophobic, slowing down the degradation^3^. Molecular weight is playing an important role in degradation, with a higher molecular weight leading to slower degradation, so promoting prolonged release of embedded drugs^4^. Especially biodegradable implants for targeted drug delivery and long-acting injectable (LAI)^5^ formulations benefit from the versatile nature of PLGA, as they can be tuned in their release behavior by engineering the polymer to improve the local drug administration. As the subsequently generated degradation products enter natural metabolic pathways, PLGA is safe for *in vivo* applications^6^. For direct targeted delivery of drugs to specific locations, a degradable drug releasing implant, placed directly at the site of action, can be advantageous^7^.

Dexamethasone (Dex) is a synthetic glucocorticoid with strong anti-inflammatory and immune-suppressive properties and has been used in various PLGA-based formulations, such as microspheres^8, 9^ or implants^10^ for ocular drug delivery^11, 12^ or intracochlear applications^13^, to achieve long-term anti-inflammatory effects while minimizing systemic side effects. PLGA formulated in microspheres containing Dex shows an initial burst release, due to the high surface area^14^, not observed for implants, which instead exhibit a lag phase in the beginning of the release period, followed by a sustained release phase with subsequent erosion of the implant^15^. To achieve increased release rates and a reduction in lag time, the more hydrophilic dexamethasone phosphate has been studied, however only minor changes in release behavior were observed^16^. Relevant parameters in tuning dexamethasone-release from PLGA-implants are the molecular weight, and the end-group capping, both relevant for PLGA-hydrolysis on the surface of the implant. With further change of size and shape of the implant, water absorption and erosion is further modifying the surface, thus resulting in variable release rates^17^.

The addition of plasticizers can alter the mechanical properties, whereby pores and cavities inside the PLGA matrix are created, thus increasing the kinetics of the degradation processes, additionally softening the material^18^. As water in particular is decisive for the degradation process for the PLGA^19^, hydrophilic polymers are particularly relevant as plasticizers to modulate water uptake and thus degradation behavior. Among these, polyethylene glycol (PEG)^20^ is one of the most widely used additives extensively employed in applications such as nanoparticles^21, 22^ and microspheres^23^, as well as implants^24^, in combination with various drugs, such as dexamethasone^24^ or paclitaxel^22^. Different strategies, such as via covalent binding^21^, known as PEGylation, as well as the creation of polymer blends^24, 25^, demonstrate its usability as additive in the field of PLGA-based drug delivery. Many clinically approved PLGA-based drug delivery systems, such as Ozurdex^®^ and Risperdal Consta^®^, exhibit suboptimal release kinetics, most notably a pronounced lag phase followed by an uncontrolled burst release phase^26, 27^. A comparison of risperidone loaded PEG-PLGA microparticles compared to commercial Risperdal Consta^®^ shows a significant optimization, as no lag phase was observed in vitro and a linear release over one month can be achieved^28^. Ozurdex^®^ is a long-acting ophthalmic implant approved by the FDA for the treatment of non-infectious uveitis, macular edema following branch or central retinal vein occlusion, and diabetic macular edema, and represents one of the most frequently administered thin PLGA-based implants. Bhagat et al.^27^ demonstrated that Ozurdex®, which contains more than 60 wt% dexamethasone, may fracture upon intravitreal insertion due to the poor mechanical properties of PLGA and the lack of plasticizers, and additionally discovered the unfavorable release kinetics of the implant formulation. Substantial efforts have been made to enhance the initial burst release of PLGA-based implants through the incorporation of various plasticizers, including PEG and polyvinyl pyrrolidone (PVP)^29^. Their incorporation resulted in a first order and pseud zeroth order release kinetic, due to the formation of an interconnected porous network inside the implant^29^. For *in-situ* forming implants, additives like crosslinked poly(acrylic acid) (Carbopol), hydroxypropyl methylcellulose (HMPC), stearic acid and acetyltributyl citrate have been explored in compositions (1%, 3%, 5%), demonstrating a strong influence on morphology and swelling behavior, though with an only limited impact on the Dex release in most cases.^30^ The incorporation of hydroxypropyl-β-cyclodextrin and PEG 8000 was tested for methoxyestradiol release from cylindrical implants, inhibiting the lag phase time, but resulting in a more pronounced burst release.^31^ In previous studies^13, 24, 32^, we investigated the impact of PEG as plasticizer onto Dex release from thin implants, discovering a reduction in lag phase, resulting in an increased release rate in the first week. However, during handling of softer implants consisting of a PLGA and PEG blend, a deformation was observed by surgical instruments^33^, resulting in a change of the cylindrical shape, which could lead to uncontrolled drug release^34^. Additionally, drawbacks of PEG have been discovered, resulting in less overall approval in the pharmaceutical field^35^, thus encouraging the research in pursuit of similar alternatives.

We here investigate alternative plasticizers with the potential to mitigate the limitations associated with PEG-based formulations. In this context, poly(2-oxazoline)s (POx) represent a promising class of materials. POx are highly tunable, biocompatible polymers with adjustable molecular architecture^36^, and have been proposed as next-generation PEG alternatives in pharmaceutical and biomedical applications^37-40^.The wide range of possible aliphatic side chains enables tuning of the polymer’s hydrophilicity. Poly(2-methyl-2-oxazoline) (PMeOx) shows an excellent water solubility, and with increasing length a lower critical solution behavior (LCST) is observable ranging from 60°C for poly(2-ethyl-2-oxazoline) (PEtOx), down to 25°C for poly(2-*N*-propyl-2-oxazoline) (PPrOx)^41^. Additionally, the length of the alkyl group influences the crystallization behavior, as the glass transition temperature is decreasing with increasing aliphatic side chain length^42^. At a length of more than five carbon atoms, the polymers start to show semi-crystalline behavior with a melting temperature of ~150°C^43^. Due to their favorable physicochemical properties, including a reduced immunogenic potential and good mechanical compatibility with polyester-based matrices, POx are attractive candidates for modifying both the release kinetics and mechanical properties of PLGA-based implants.

We here probe the blending of different poly(2-alkyl-oxazoline)s as plasticizers into PLGA, with a focus on physical and mechanical properties, phase separation behavior and the subsequent dexamethasone-release. The blending of polymers with progressively longer alkyl side chains (‑methyl, ‑ethyl, ‑propyl, ‑butyl), thereby increasing hydrophobicity, is proposed to impact the miscibility behavior of the polymer blend, resulting in different phase separation with an impact in the embedded dexamethasone’s drug release kinetics.

2. Materials and Methods

2.1. Materials

PLGA (Expansorb DLG 50-2A) was purchased from *seqens* (Lyon, France). Methyl oxazoline was purchased from *abcr* (Karlsruhe, Deutschland). Dexamethasone was acquired from *Caesar & Loretz GmbH* (Hilden, Germany). All other chemicals were purchased from *merck* (Darmstadt, Germany). Dry acetonitrile (CAN) was taken from a solvent purification system (mB-SPS-5, MBRAUN, Garchingen, Germany).

2.2. Synthesis

2.2.1. General Procedure

The oxazoline monomers (2-methyl-, 2-ethyl-, 2-propyl- and 2-butyl-1,3-oxazoline) were freshly distilled prior to each reaction over barium oxide, BaO, and stored under inert conditions in a glovebox. The reaction mixtures were prepared under inert conditions in the glovebox, then transferred to a microwave reactor. The reactions were executed with a wattage of 50 W at a temperature of 100°C in 30 min, subsequently quenched with water over night and then precipitated in cold (0°C) diethyl ether. The resulting polymers were then dried at elevated temperatures, yielding a white powder.

2.2.2. Exemplary Procedure for Methyl Oxazoline

Under inert conditions dry acetonitrile, ACN (1 mL), freshly distilled 2-methyl-1,3-oxazoline (1 mL, 12.2 mmol) and recrystallized methyl tosylate (84 mg, 0.45 mmol) were added to the microwave tube, equipped with a stirring bar and a microwave septum. The vial was transferred from the glove box to the reactor and was subsequently exposed to a wattage of 50 W at a temperature of 100°C for 30 min. The mixture was cooled down to 50°C, then removed from the microwave reactor and afterwards quenched with distilled water (0.1 mL) over a period of 16 h. The polymer solution was then precipitated in 10-fold excess of cold (0°C) diethyl ether twice and dried under reduced pressure at 60°C, to yield (> 95%) a white powder. All other polymers were conducted in a similar manner, using the respectively different monomers.

2.3. GPC

Gel permeation chromatography was performed at 30°C on a Viscotek GPCmax VE 2001 (Malvern Panalytical, Great Malvern, England) equipped with a CLM3008 precolumn and a GMHHR-N-18055 main column in a column Heater at 60°C. As solvent DMF with 100 mM LiTf_2_N was used. The sample concentration was 3 mg/mL while applying a flow rate of 1 mL/min. The refractive index detection was performed with a VE 3580 RI detector of Viscotek^TM^. For determination of the molecular weights, external calibration was done using polystyrene (PS) standards with a molecular weight range from 1.050 to 170.000 g/mol. OmniSEC software (Version 5.12.) was used for evaluating data.

2.4. MALDI-TOF

Matrix-assisted laser desorption/ionization mass spectrometry measurements were performed on a Bruker autoflex maX^TM^ MALDI TOF/TOF System (Bruker Daltonics, Billerica, MA, USA) using a BRUKER smartbeam^TM^-II nitrogen laser, operating at a wavelength of 355 nm. The used matrix:analyte:salt ratio was 3:3:1. The polymer samples were dissolved in THF or HFIP with a concentration of 2 mg/mL. Dithranol was used as matrix with a concentration of 20 mg/mL in THF and NaTFA was used as salt with a concentration of 2 mg/mL in THF. Data evaluation was carried out using the flexAnalysis software (3.4) and simulation of the isotopic pattern was performed by Data Analysis software (version 4.0).

2.5. DSC

Differential scanning calorimetry (DSC 5+ STAR^e^System, Mettler Toledo, Greifensee, Germany) was used to investigate thermal transitions. Samples were heated from -40°C to 70°C, using a heating rate of 20 K/min in a nitrogen atmosphere (40 mL/min). Data were collected in the second heating cycle. Data analysis was performed on the STAR^e^ Software (version 19.00c) and Origin 2019 (OriginLab Corporation).

2.6. Rheology

Rheological measurements were performed on Anton Paar MCR-702-DSO rheometer (Anton Paar, Graz, Austria) equipped with parallel plate-plate geometry (d = 8 mm). The device was equipped with Peltier-temperature control (P-PTD 220), ensuring accurate temperature control and nitrogen gas flushing. Samples were prepared from solution (acetone) and dried in a vacuum oven for 48 h at elevated temperatures. Before each measurement, the samples were held at a target temperature for at least 10 min to ensure equal temperature distribution. Recorded data were analyzed *via* RheoCompass^TM^.

2.7. WAXS

Wide angle x-ray scattering was performed at room temperature using a Retro-F laboratory setup (SAXSLAB, Copenhagen, Denmark) equipped with a microfocus X-ray source (AXO Dresden GmbH, Dresden, Germany) and ASTIX multilayer X-ray optics (AXO Dresden GmbH) as monochromator, resulting in CuKα radiation with a wavelength of 0.154 nm. The diameter of the circular beam was about 1.5 mm and the exposure time was 180 s. The scattered X-ray intensity was recorded by a two-dimensional PILATUS3 R 300K detector (DECTRIS Ltd., Baden, Switzerland), with the sample-to-detector distance of about 85 mm.

2.8. E^-^-beam Sterilization

Electron beam irradiation was selected as the sterilization method. The extruded material was cooled on ice and subsequently irradiated with a total dose of 25 kGy using a 10 MeV linear accelerator (MB 10–30 MP, Mevex, Stittsville, Ontario, Canada). Irradiation was performed on a moving tray at a speed of 95 cm/min. The accelerator operated at a repetition rate of 460 Hz with pulse durations of 8 µs, employing a scanning frequency of 3 Hz and a scanning width of up to 60 cm. The total dose of 25 kGy was delivered in two separate doses of 12.5 kGy each.

2.9. Implant preparation using hot-melt extrusion

PLGA was pulverized using a cryo-mill (Retsch GmbH, Haan, Germany) for four cycles of 90 s at 25 Hz. The resulting polymer powder was mixed with poly(2-oxazoline)s and dexamethasone and subjected to a second cryo-milling step under milder conditions, using one cycle of 90 s at 15 Hz. This homogeneous powder was subsequently used for hot-melt extrusion, which was conducted using a ZE 5 ECO twin-screw extruder (Three-Tec GmbH, Seon, Switzerland) equipped with a 0.3 mm die, operated at a screw speed of 80 rpm. The three heating zones were maintained at 76°C, 76°C, and 80°C (from the feeding zone to the die). The extrudates were manually cut into 10 cm segments and stored at −20°C until further analysis.

2.10. In vitro drug release studies

Drug release studies of dexamethasone were performed in a phosphate buffered saline (PBS) at pH 7.4 at a temperature of 37°C and 40 rpm over a duration of 8 weeks. After each sample taken, the solvent was completely exchanged, to ensure sink conditions. Each sample was measured in triplets. Analysis was done by high-performance liquid chromatography (HPLC). Experiments were conducted on a VWR HITACHI Chromaster HPLC (Avantor, Radnor, PA, USA), using a InfinityLab Poroshell 120 EC-C8 (4.6 x 250 mm) column with a InfinityLab Poroshell 120 HILIC (2.1 x 5 mm) UHPLC guard. A mixture of ACN, water, and formic acid (60:40:0.1) was used as a solvent. The detection wavelength was set to 242 nm.

2.11. Optical microscopy

Cross-polarized light microscopy was performed using a Leica DMRXE (Leica, Wetzlar, Germany) upright transmitted-light microscope, which was equipped with a 10× Plan objective with a numerical aperture of 0.20. Images were recorded at a spatial resolution of 2080 × 1544 pixels using the microscope’s integrated halogen illumination system, with the built-in analyzer-polarizer configuration generating crossed-polar conditions.

2.12. X-ray microscopy

X-ray microscopy was performed using a ZEISS Xradia 810 Ultra (ZEISS X-ray Microscopy, Jena, Germany), which operates at a quasi-monochromatic photon energy of 5.4 keV (Cr). The sample was mounted on the tip of a metallic pin and inserted in the sample holder. Tomographic datasets were acquired over a 180° angular range in Large Field of View (LFOV) mode with camera binning 2 (field of view 65 µm; voxel size 128 nm) using Zernike phase contrast. Projection images were reconstructed into a 3D tomographic volume using the ZEISS reconstruction software (XMReconstructor). Reconstructed volume was then exported as a stack of 16-bit tiff images for vizualization in Avizo3D (Thermo Fisher).

2.13. Texture analysis

Mechanical resistance of the extruded formulations was evaluated on a CT3 Texture Analyzer (Ametek GmbH, Hadamar-Steinbach, Germany), equipped with a blade (accessory TA7, knife edge, Ametek GmbH, Germany), operated over a maximum distance of 0.2 mm at a constant velocity of 0.01 mm/s. The trigger force was set to 0.02 N. All experiments were performed at 20°C. For data evaluation the TexturePro CT V1.6 software was used. For each sample 15 measurements were taken, followed by a statistical evaluation.

3. Results and Discussion

The aim of this study was to investigate the influence of poly(2-alkyl-oxazoline)s with different side chains on the mechanical properties of a known PLGA formulation with dexamethasone, as well as the therefrom resulting drug release kinetics. Specimens were prepared from a solid, cryo-milled mixture, then extruded, using a twin-screw extruder, to provide uniform shape and diameter, as well as even distribution throughout the whole implant. The mechanical properties were studied using a texture analyzer as well as melt rheology to study the behavior before, during and after the extrusion, to gain insight in the hypothesized plasticizing effect.

3.1. Polymer synthesis

2-Alkyl-1,3-oxazolines were polymerized using cationic ring opening polymerization^44^, using methyl tosylate as an initiator^45^ to reach control over their chain length and endgroup functionalization. Different monomers bearing methyl- (MeOx), ethyl- (EtOx), propyl- (PrOx) and butyl- (BuOx) side chains were prepared, with a projected molecular weight (Mn ~ 2000 g/mol) adjusted by the monomer/initiator ratios, see Table 1. The desired low molecular weights prospect an improved miscibility with the PLGA polymers. Reactions were carried out in a microwave reactor due to its better energy transfer to allow fast initiation^46^. Under inert conditions, 1 mL of acetonitrile, ACN, 1 mL of monomer as well as a corresponding amount of initiator were added to the microwave tube, to reach the target molecular weight of 2000 g/mol, resulting in four polymers with different side chains, with a narrow molecular weight distribution and PDIs less than 1.1, see Figure 1. The resulting polymers are listed in Table 1. Polymers were further analyzed using matrix-assisted laser desorption/ionization time of flight mass spectrometry (MALDI-tof MS), where for all polymers a clean sodium series was observed, see Figure 2a and Figure S1-S3. The main series of the peaks in the MALDI-TOF spectrum are matching with the simulated spectra, including isotopic patterns, so proving chemical identity of the polymers, and livingness of the polymerization by presence of the initiating methyl tosylate and the terminal OH-group as end groups. Figure 2b is further depicting the measured distance of 99.07 Da in between two peaks, which is matching the molecular weight of the monomeric unit, EtOx. For PMeOx (Figure S1) and PEtOx (Figure 2) an additional series was observed, with lower molecular weight, stemming from a proton initiated side product, occurring due to the more hydroscopic monomers.

Figure 1: a) Schematic representation of the sythetic pathway for the synthesis of the poly(2-alkyl-oxazoline)s *via* living cationic polymerization b) chemical structures of the synthesized poly(2-methyl-oxazoline) (PMeOx), poly(2-ethyl-oxazoline) (PEtOx), poly(2-*N*-propyl-oxazoline) (PPrOx), poly(2-*N-*butyl-oxazoline) (PBuOx).

Table 1: Composition and GPC results of the prepared POx polymers (Mn, Mw, PDI).

| Monomer | Polymer^a)^ | [M]/[I]-ratio | M_n_ (GPC) | M_w_ (GPC) | PDI |
| --- | --- | --- | --- | --- | --- |
| Methyl oxazoline | PMeOx | 23.5 | 2000 | 2100 | 1.06 |
| Ethyl oxazoline | PEtOx | 20 | 2100 | 2300 | 1.09 |
| n-Propyl oxazoline | PPrOx | 17.7 | 2100 | 2150 | 1.05 |
| n-Butyl oxazoline | PBuOx | 15.7 | 2000 | 2100 | 1.06 |

1. synthesis in dry ACN, microwave, 100°C, 50 W, 30 min

3.2. Sterilization and preparation of filaments *via* hot-melt extrusion

For further use in medical applications, e.g. animal testing, the polymers were subjected to electron-beam sterilization. Critical herein is to check their stability, and the need to exclude chemical changes during the electron beam sterilization. Thereto the polymers are irradiated with a total dose of 25 kGy, delivered in two doses of 12.5 kGy each, thus ensuring safety for future *in vivo* tests. The analysis with GPC and MALDI revealed negligible changes before and after the sterilization. Figure 2b shows a comparison of the polymers before (red) and after sterilization (green), recorded *via* MALDI-Tof-MS. As the molecular weights are not changing, we conclude, that the chemical structure is still fully intact after sterilization, as no new series are detected. Additionally, the introduced end groups (-CH_3_ and -OH) are still attached, so proving their excellent stability. In addition, molecular weights are remaining unchanged as proven by SEC, with no increase of the PDIs (Figure 2c), allowing to conclude that no major decomposition process are taking place during this sterilization process. Figure S1, S2 and S3 depict the same results for the corresponding polymers PMeOx, PPrOx and PBuOx.


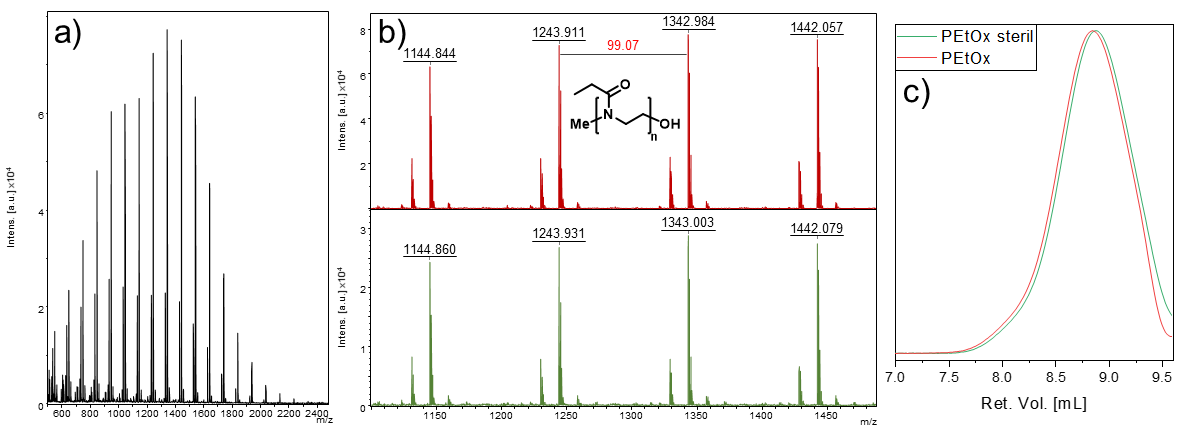


Figure 2: a) Full MALDI-tof-MS spectrum of PEtOx; b) detailed MALDI-tof analysis of PEtOx before (red) and after (green) sterilization. The red values indicate the distance between the signals matching the monomeric units; c) GPC results of PEtOx before (red) and after (green) sterilization.

Table 2: detailed compositions of the extruded formulations. The different oxazoline species are specified in brackets (M: PMeOx, E: PEtOx, P: PPrOx, B: PBuOx, Dex: dexamethasone).

| Sample | PLGA content (wt%) | POx content (wt%) | Dex content (wt%) |
| --- | --- | --- | --- |
| M10 | 80 | 10 (methyl) | 10 |
| M20 | 70 | 20 (methyl) | 10 |
| E10 | 80 | 10 (ethyl) | 10 |
| E20 | 70 | 20 (ethyl) | 10 |
| P10 | 80 | 10 (propyl) | 10 |
| B10 | 80 | 10 (butyl) | 10 |

All formulations were prepared from PLGA *via* cryo-milling, first creating a fine powder, followed by addition of different POx powders and dexamethasone (Table 2), to create a homogenous physical powdery mixture. The mixtures were further processed using hot-melt extrusion with a twin-screw extruder, in order to create smooth extrudates with a uniform diameter of 0.3 mm and an even distribution of dexamethasone and POx. All samples displayed a smooth surface and were extruded without complications. Formulations containing 20 wt% of propyl oxazoline or butyl oxazoline were also probed, but were already demixing from the PLGA during the hot-melt extrusion process and were therefore not pursued any further.

3.3. Mechanical analysis

For implantation, the mechanical properties of polymer-based implants are critical to enable reliable handling and placement during surgery. Plasticizers such as PEG are therefore commonly incorporated into PLGA, since neat PLGA implants are often too stiff and brittle^47^. Because plasticization typically lowers the mechanical strength^48^, it can reduce the likelihood of intraoperative fracture and the formation of sharp edges. The impact of blending PLGA with POx on the physical properties of the so generated polymer blend was therefore analyzed using rheological measurements, investigating the behavior of PLGA mixtures with the synthesized POx at different temperatures, e.g. 30°C, 50°C and 70°C. The storage modulus G’ and the loss modulus G’’ were measured to determine the stiffness of the material at different temperatures, as shown in Figure 3a-f. In comparison to virgin PLGA, see Figure S4, a reduction in stiffness was observed for most samples, except for M20, E10 and E20. The greatest impact was observed for P10, Figure 3e, with the addition of n-propyl oxazoline, where a change of one order of magnitude was observed, in comparison to the virgin PLGA. Sample P10 was the only sample, that could be measured at 30°C, whereas pure PLGA and all other mixtures were too stiff under the chosen measuring conditions. Blends with higher POx content show an opposing trend, where for sample M20, the viscosity is drastically increased at 50°C, while for E20 the viscosity is slightly reduced, when compared to the blend E10, containing a reduced amount of POx. Interestingly, most prepared mixtures show a shear thinning behavior, which can be a beneficial trait for hot-melt extrusion, as it enables the handling at lower temperatures, due to the shear induced reduction in viscosity.

To compare the samples in view of their mechanical strength, the extruded formulations were tested in a texture analyzer to assess the structural integrity and penetration resistance of the implants (Figure 4). This test allowed determination of whether the implants break upon loading or whether the blade was able to penetrate without inducing damage. The addition of a plasticizer softens the material and thus the penetration depth before sample rupture should increase. For samples M10 and M20, the required force to reach the breaking point is reduced, which indicates, that the desired plasticizing effect of the added methyl oxazoline is less than the loss in mechanical stability provided by the PLGA matrix itself. Extrudates E10, P10 and B10 show a similar mean force resistance, comparable with PLGA. Sample E20 proves to be the most resistant of all the samples, with a mean breaking force of 2.4 N at a penetration depth of 0.9 mm, thus revealing the most promising properties. The incorporation of the different POx shows predominantly no significant effect on the mechanical resistance behavior. However, as the tests were performed at room temperature, the extrudates will perform differently und body temperature conditions.


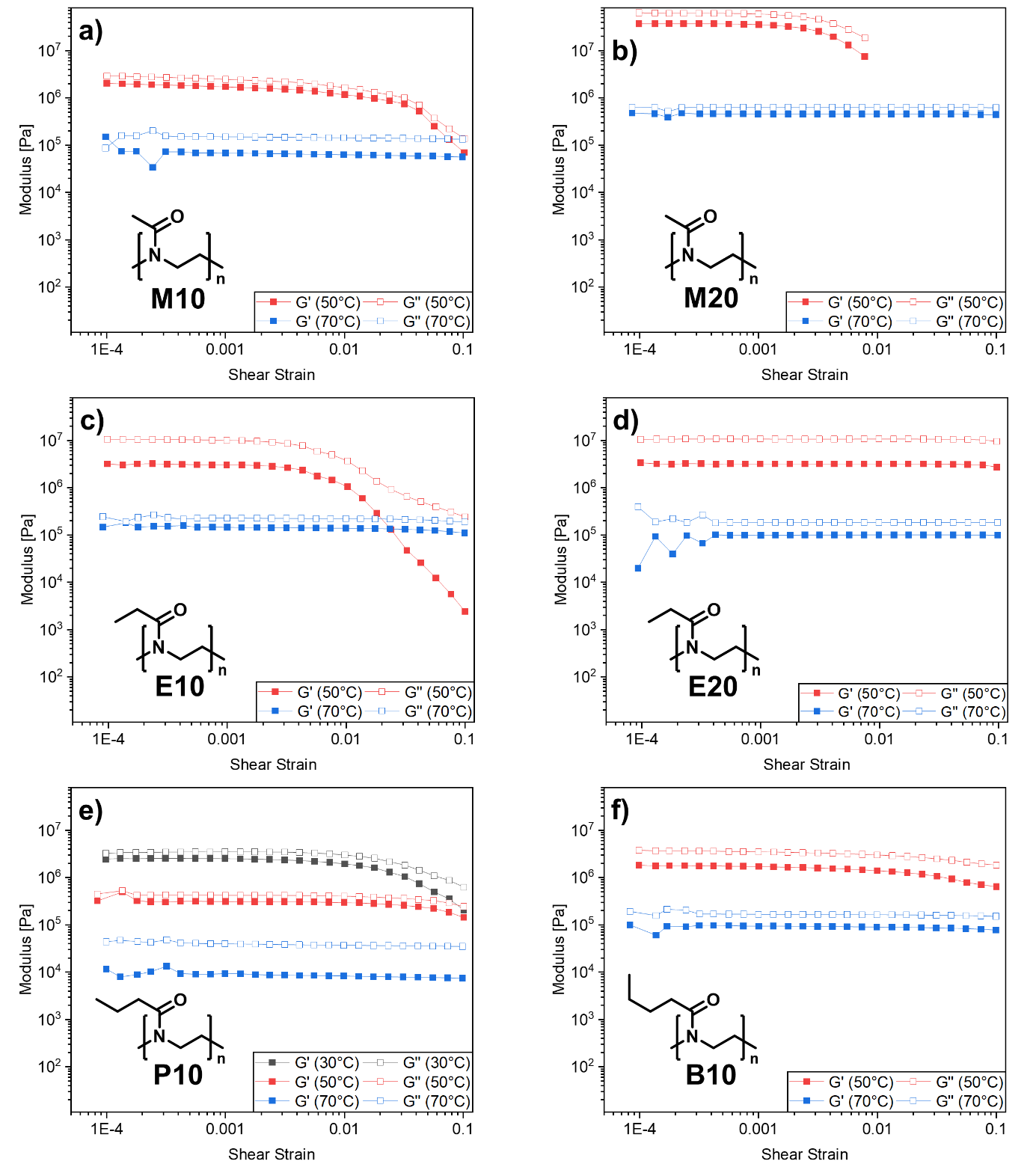


Figure 3: Rheological investigation of PLGA blends: a) M10, b) M20, c) E10, d) E20, e) P10, f) B10, with POx (10 wt% and 20 wt%) at different temperatures (30°C, 50°C, 70°C).


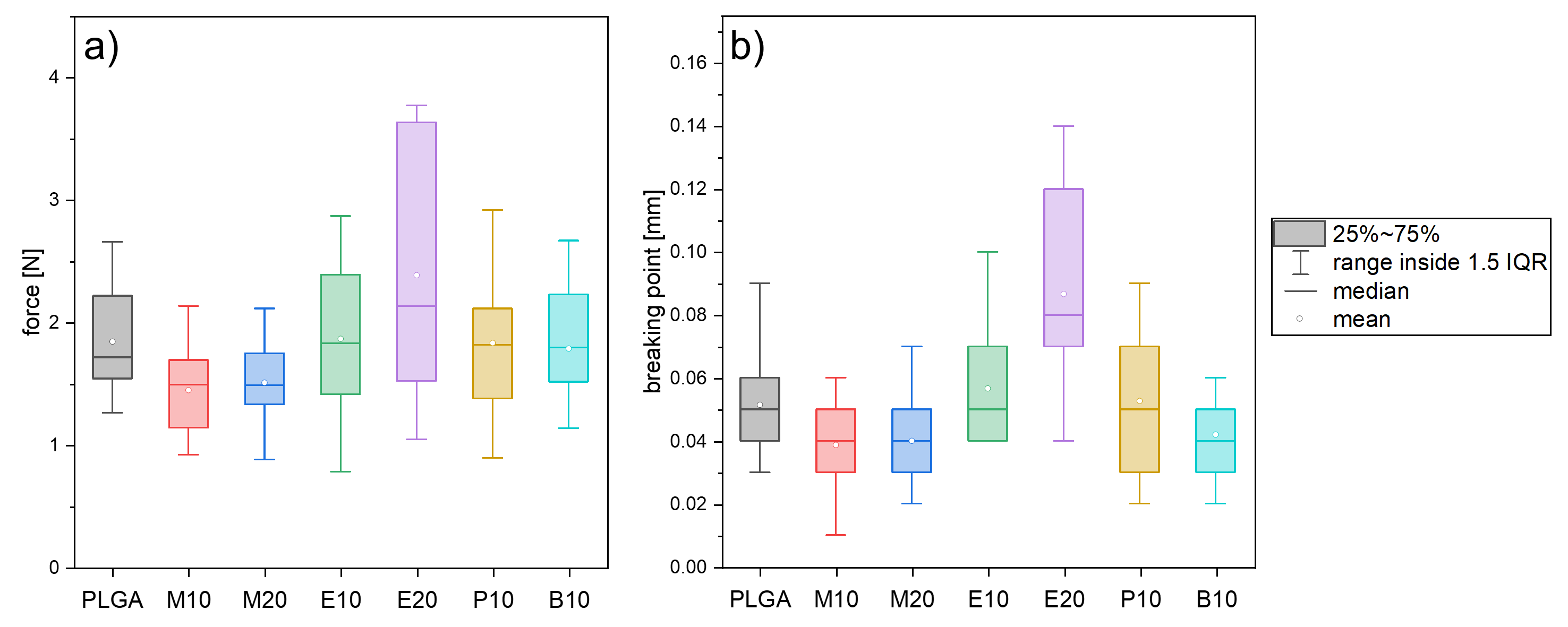


Figure 4: Statistical results from the texture analysis, performed with a knife blade, cut in a 90° angle at a speed of 0.01 mm/s showing a) the breaking force required and b) the penetration depth as the filament broke.

3.4. Morphological and Structural Characterization of Extruded Filaments

Optical microscopy (OM) was used to examine the surface of the extruded filaments and determine their exact diameter, revealing a mean diameter of 370 µm (Figure 5). As the nozzle diameter of the extruder was 300 µm, warping of the specimen was observed during the extrusion process. Due to the shear force that the formulation experiences during the extrusion process and its viscoelastic behavior, it expands after passing the nozzle. The images show a smooth surface for all samples, suggesting no mayor phase separation. Additionally, the three-dimensional X-ray microscopy images of the extrudate M10 (Figure S5) show no discernible phase separation or other morphological heterogeneities at the available resolution (pixel size 128 nm), indicating that any phase separation is either absent or occurs at length scales below the effective detection limit of the measurement (i.e., features smaller than ~500 nm). From the taken OM images, the gray values were determined. From these values the “whiteness” can be determined, therefore a conclusion for smoothness can be drawn. As the full surface could not be analyzed, spot checks were performed. For all samples, the gray value is above 200 with only small deviations, decreasing at the edges, due to the round cross section profile of the specimen. In rare occasions, also larger deviations were observed, most likely stemming from larger crystalline aggregates of dexamethasone on the surface.

We then put our attention to the miscibility of the POx inside the PLGA via their thermal properties. If thermal transitions of both polymers would change, an at least partial miscibility could be derived; whereas fully separated thermal transitions, identical to the pure homopolymers, would indicate an immiscible polymer blend. DSC measurements revealed no significant changes in the glass transition temperatures, T_G_ of the PLGA after the incorporation of the POx and dexamethasone after extrusion (Figure S6), in comparison to neat PLGA. However, the appearance of a second transition, a T_G_ at lower temperatures was observed for samples M20 and E20, caused by addition of the PMeOx and PEtOx respectively. The observed T_G_’s for the virgin polymers were at the temperatures T = 20.9°C for methyl oxazoline and 0.8°C for ethyl oxazoline, whilst the observed T_G_’s in the formulations are at 12.9°C and 14.7°C respectively. This is indicating an at least partial miscibility of PLGA with the POx. Similar observations were made using XRD (Figure 6). The formulated samples show a wide amorphous halo, originating from the amorphous PLGA matrix at 1.5 Å^‑1^. For all samples, the reflexes of the incorporated dexamethasone could be observed, concluding that the drug is dispersed in the polymer matrix and small crystallites are still remaining, similar to the findings from the optical microscopy, an observation frequently made in comparable literature^49, 50^. For samples P10 and B10, a second amorphous halo was observed in the range of 0.4 Å^-1^. When compared to the virgin polymers, phase separation is assumed, as a second amorphous halo in the same range is observed, proposedly originating from the interactions of the side chains with the x-ray beam. The comparison of the extrudates with a mixture of PLGA and the oxazoline polymers, taken from solution and dried under reduced pressure, shows no significant difference, hence demonstrating no impact of the extrusion process on the phase development of the polymer blend.


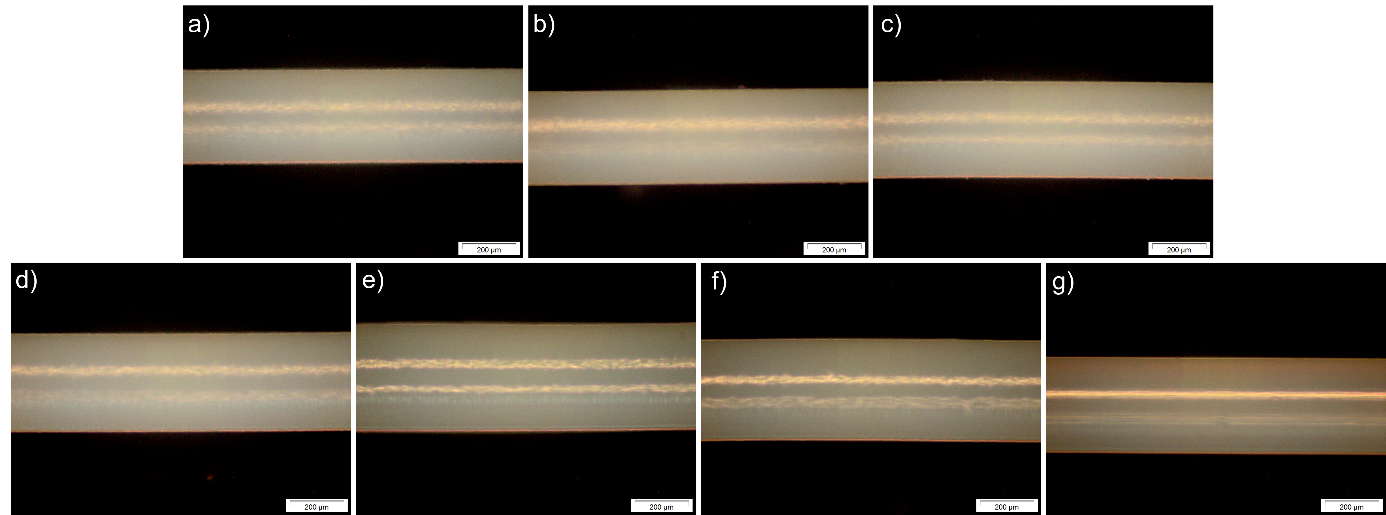


Figure 5: Optical microscopy pictures of the extruded filaments; a) PLGA with dexamethasone 10 wt%, b) M10, c) M20, d) E10, e) E20, f) P10, g) B10 in crossed polarized light. Scale bars are 200 µm.


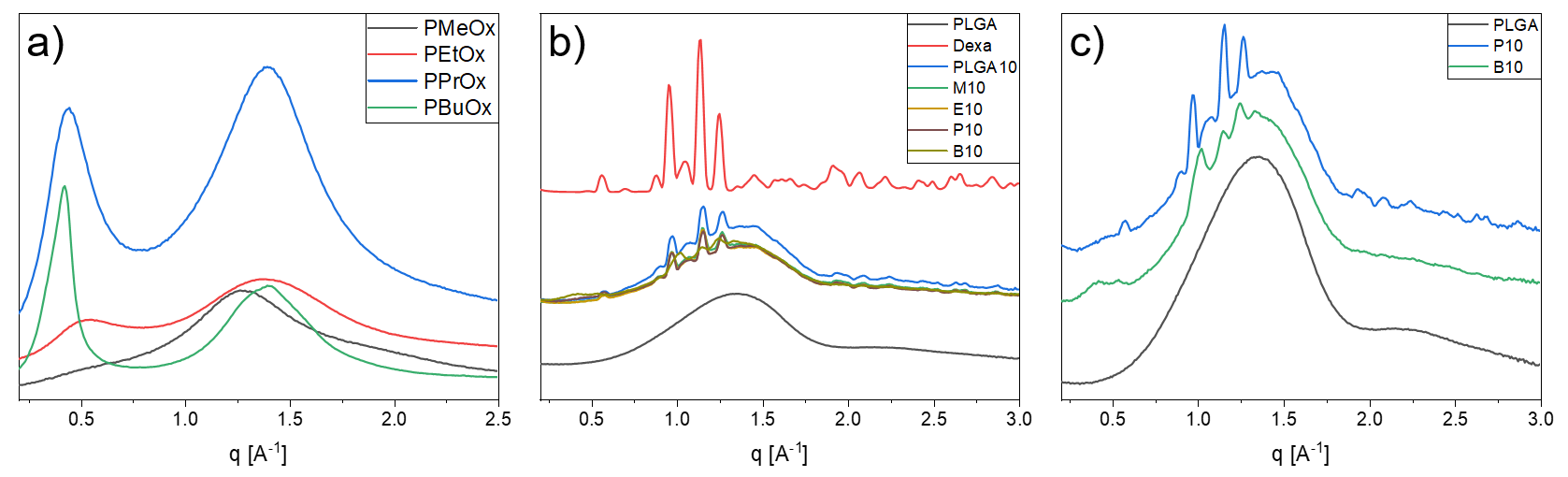


Figure 6: Wide angle x-ray measurements of a) the virgin POx; b) the extruded formulations, compared to virgin PLGA and dexamethasone; c) a closer depiction of P10 and B10 compared to virgin PLGA.

3.5. *In vitro* drug release studies

Figure 7 shows the observed dexamethasone release kinetics from extruded formulations of PLGA and the POx with differently polar side chains loaded with 10 wt% dexamethasone. All release profiles were taken as triplets. The different formulations were submerged in an aqueous phosphate buffered saline (PBS) at pH 7.4 for a duration of 8 weeks, to investigate the impact of the added polymers. PLGA formulations tend to show an observable lag phase in the first week, which was less pronounced in the case for PLGA Expansorb 50-2A, as similar observations were made in preceded publications^51, 52^. For samples E10, E20 and P10 a lag phase of 5 days was observed with a cumulative release of less than 5 wt% dexamethasone released. Samples M10, M20 and B10 exhibit a shorter lag phase of 3 to 4 days, with a similar amount of dexamethasone released in this period. These three samples also show a faster drug release, 80 wt%, 97 wt% and 91 wt% respectively after 20 days, compared to the native PLGA formulation, which shows a release of 44 wt% after 20 days, similar to the other polymer mixture based formulations E10, E20 and P10. These display a more similar release profile to PLGA, with a cumulative release of approximately 90 wt% dexamethasone released after the studied period of 8 weeks. Extrudate M20 already showed a completed release after 21 days, similar to B10, where a release window of 24 days was observed. For sample M10 a comparable release behavior compared to M20 was observed for the first 8 days, followed by a decrease in release speed, showing a release of 90 wt% drug released after a period of 27 days. It can be concluded that the differences in drug release behavior stem from the different nature of the poly oxazolines added. The addition of the more hydrophilic PMeOx is enhancing water uptake, due to its faster dissolving nature, thus increasing the surface area and so promote the release of the embedded dexamethasone. PEtOx and PPrOx show a partial miscibility with PLGA due to their less polar nature, when compared to PMeOx. As their water solubility is limited, no significant change in release behavior was observed.

Compared to previously performed studies regarding PEG as plasticizer for PLGA, in blends or as co-polymers of similar filaments^13, 16, 32^, the here presented release kinetic shows no initial burst release and a shorter lag time. For PLGA blends containing PEG 1500 a release after 4 days of 40 wt% Dex release for higher PEG contend (15 wt%) and 20 wt% Dex release for lower PEG contend (10 wt%) was observed^13^. Compared to the here presented formulations, which are showing a maximum of 9 wt% Dex release after 4 days, this is an improvement of the otherwise uncontrolled release. The initial lag phase we observed for samples M10, M20 and B10, with a range of 3 to 4 days, shows to be shortened, when compared to PEG-PLGA co-polymer matrices, which show an initial lag phase of 10 days^32^, which were similarly observed for dexamethasone phosphates^16^. Additionally, the in here discussed formulations with POx, E10, E20 and P10, show a more sustained release over the release period, with a reduced lag phase of 5 days, which is a significant improvement for the sustained dexamethasone delivery.


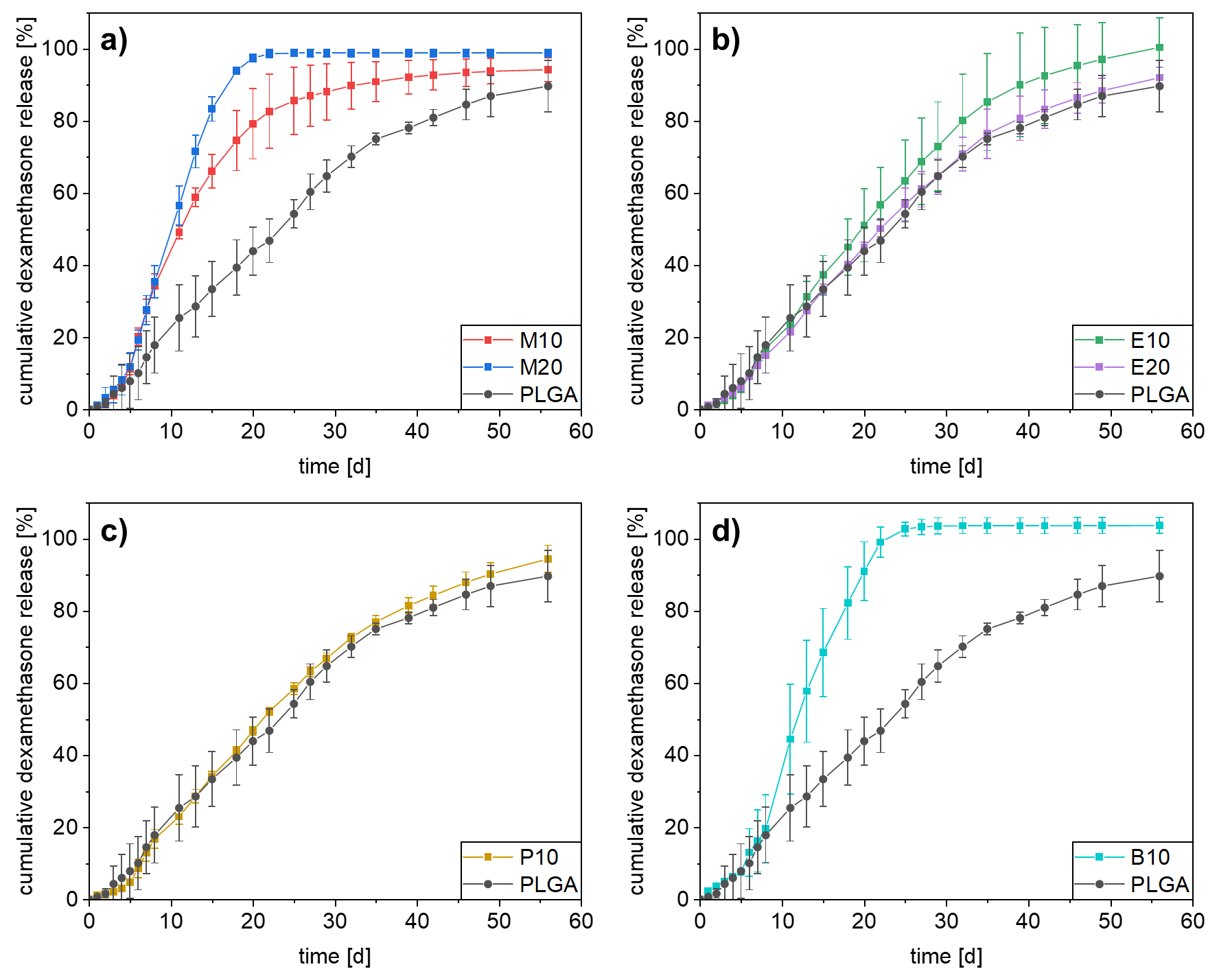


Figure 7: Drug release study performed in PBS (pH 7.4) at 37°C in a shaker set to 40 rpm from all extrudates; a) formulations with PMeOx; M10 and M20, compared to PLGA, b) formulations with PEtOx; E10 and E20, compared to PLGA, c) formulation with PPrOx; P10, compared to PLGA, d) formulation with PBuOx; B10, compared to PLGA. All tests were performed in triplets, here displayed with corresponding error bars.

4. Conclusion

Different poly(2-alkyl-oxazoline)s were successfully synthesized using microwave induced living cationic ring opening polymerization, with low molecular weights (2 kDa) and excellent PDI’s (< 1.1), as proven via GPC. MALDI-tof-MS proved the presence of the initiating groups, indicative of the livingness of the polymerization. To demonstrate the potential of poly(2-alkyl-oxazoline)s as a additive for PLGA in drug delivery applications, the synthesized polymers were blended into a PLGA matrix (10 wt%/ 20 wt%) and subsequently formulated with dexamethasone (10 wt%) using cryo-milling and hot-melt extrusion. The prepared formulation with PPrOx (P10) shows a significant reduction in viscosity, as measured with melt rheology, when compared to the virgin PLGA, indicating a softening effect, whilst at the end retaining a similar release profile to comparable Dex containing PLGA filaments, over the whole release period. Samples containing PMeOx, PBuOx (M10, M20 and B10) all show an increased release rate of the dexamethasone, together with a reduction in the lag time (t_lag_ = 72 h), coming with a slight reduction in mechanical resistance. In comparison to previously mentioned similar PLGA formulation containing PEG 1500 and Dex, no initial burst release was observed^13^, which is seen as a significant advance. Similarly prepared extruded filaments containing a PLGA-PEG co-polymer matrix, formulated with Dex, show an extended lag phase, in comparison to the here presented formulations^32^. Compared to pure PLGA, samples with added PEtOx, E20, shows a good plasticizing effect. Even though the glass transition, T_G_, is not changing significantly, the mechanical stability and plasticity are significantly improved, hence increasing the rupturing force required to fracture the extrudate. In addition, also the rheological profile shows a slight decrease in viscosity at the measured temperatures. At the same time, the release kinetics are comparable to comparable Dex containing PLGA filaments, showing a similar release profile over the course of an 8 week release period. This leads to the conclusion, that poly(2-alkyl-oxazoline)s can be used as plasticizers for PLGA. The possibility of different side chains and variable polymer blend compositions enables tunable properties, observed as acceleration in dexamethasone release after the addition of PMeOx and PBuOx and improvement in mechanical properties for PEtOx and PPrOx. Release of the dexamethasone is mainly guided by the water solubility of the POx-polymers, with PMeOx being the easiest to dissolve, and thus displays the fastest release. Hence the addition of POx containing blends is advantageous to tune softness and Dex-release properties of PLGA-polymers, useful for extruded medical devices, such as intracochlear implants.

Acknowledgements

We thank the preparatory excellence cluster PoliFaces of the Land Sachsen Anhalt for financial support.

References

1. Cai, H. H.; Li, A. X.; Qi, F.; Liu, R. M.; Tang, X. M.; Li, D.; Gu, Y. W.; Liu, J. Y., Application of biodegradable microsphere injections: an anticancer perspective. *Mater Adv* **2024,** *5* (8), 3094-3112.

2. Dandamudi, M.; McLoughlin, P.; Behl, G.; Rani, S.; Coffey, L.; Chauhan, A.; Kent, D.; Fitzhenry, L., Chitosan-Coated PLGA Nanoparticles Encapsulating Triamcinolone Acetonide as a Potential Candidate for Sustained Ocular Drug Delivery. *Pharmaceutics* **2021,** *13* (10), 1590.

3. Skidmore, S.; Hadar, J.; Garner, J.; Park, H.; Park, K.; Wang, Y.; Jiang, X. J., Complex sameness: Separation of mixed poly(lactide-co-glycolide)s based on the lactide:glycolide ratio. *J Control Release* **2019,** *300*, 174-184.

4. Kohno, M.; Andhariya, J. V.; Wan, B.; Bao, Q.; Rothstein, S.; Hezel, M.; Wang, Y.; Burgess, D. J., The effect of PLGA molecular weight differences on risperidone release from microspheres. *Int J Pharm* **2020,** *582*, 119339.

5. Muddineti, O. S.; Omri, A., Current trends in PLGA based long-acting injectable products: The industry perspective. *Expert Opin Drug Deliv* **2022,** *19* (5), 559-576.

6. Semete, B.; Booysen, L.; Lemmer, Y.; Kalombo, L.; Katata, L.; Verschoor, J.; Swai, H. S., In vivo evaluation of the biodistribution and safety of PLGA nanoparticles as drug delivery systems. *Nanomedicine* **2010,** *6* (5), 662-71.

7. Bassand, C.; Freitag, J.; Benabed, L.; Verin, J.; Siepmann, F.; Siepmann, J., PLGA implants for controlled drug release: Impact of the diameter. *Eur J Pharm Biopharm* **2022,** *177*, 50-60.

8. Zolnik, B. S.; Burgess, D. J., Evaluation of in vivo-in vitro release of dexamethasone from PLGA microspheres. *J Control Release* **2008,** *127* (2), 137-45.

9. Hickey, T.; Kreutzer, D.; Burgess, D. J.; Moussy, F., Dexamethasone/PLGA microspheres for continuous delivery of an anti-inflammatory drug for implantable medical devices. *Biomaterials* **2002,** *23* (7), 1649-56.

10. Wachowiak, S.; Danede, F.; Willart, J. F.; Siepmann, F.; Siepmann, J.; Hamoudi, M., PLGA implants for controlled dexamethasone delivery: Impact of the polymer chemistry. *J Drug Deliv Sci Tec* **2023,** *86*, 104648.

11. Bode, C.; Kranz, H.; Siepmann, F.; Siepmann, J., In-situ forming PLGA implants for intraocular dexamethasone delivery. *Int J Pharm* **2018,** *548* (1), 337-348.

12. Saraf, I.; Kushwah, V.; Alva, C.; Koutsamanis, I.; Rattenberger, J.; Schroettner, H.; Mayrhofer, C.; Modhave, D.; Braun, M.; Werner, B.; Zangger, K.; Paudel, A., Influence of PLGA End Groups on the Release Profile of Dexamethasone from Ocular Implants. *Mol Pharm* **2023,** *20* (2), 1307-1322.

13. Lehner, E.; Gundel, D.; Liebau, A.; Plontke, S.; Mader, K., Intracochlear PLGA based implants for dexamethasone release: Challenges and solutions. *Int J Pharm X* **2019,** *1*, 100015.

14. Siepmann, J.; Siepmann, F., Release mechanisms of PLGA-based drug delivery systems: A review. *Int J Pharm X* **2025,** *10*, 100440.

15. Siepmann, J.; Siepmann, F., Modeling of diffusion controlled drug delivery. *J Control Release* **2012,** *161* (2), 351-62.

16. Lehner, E.; Trutschel, M. L.; Menzel, M.; Jacobs, J.; Kunert, J.; Scheffler, J.; Binder, W. H.; Schmelzer, C. E. H.; Plontke, S. K.; Liebau, A.; Mader, K., Enhancing drug release from PEG-PLGA implants: The role of Hydrophilic Dexamethasone Phosphate in modulating release kinetics and degradation behavior. *Eur J Pharm Sci* **2025,** *209*, 107067.

17. Borrelli, M. A.; Warunek, J. J. P.; Ravikumar, T.; Balmert, S. C.; Little, S. R., End group chemistry modulates physical properties and biomolecule release from biodegradable polyesters. *J Mater Chem B* **2025,** *13* (34), 10621-10634.

18. Steele, T. W.; Huang, C. L.; Widjaja, E.; Boey, F. Y.; Loo, J. S.; Venkatraman, S. S., The effect of polyethylene glycol structure on paclitaxel drug release and mechanical properties of PLGA thin films. *Acta Biomater* **2011,** *7* (5), 1973-83.

19. Levine, H.; Slade, L., Water as a plasticizer: physico-chemical aspects of low-moisture polymeric systems. *Water Sci* **1988,** *3* (1), 79-185.

20. Huang, C. L.; Steele, T. W. J.; Widjaja, E.; Boey, F. Y. C.; Venkatraman, S. S.; Loo, J. S. C., The influence of additives in modulating drug delivery and degradation of PLGA thin films. *Npg Asia Mater* **2013,** *5* (7), e54-e54.

21. Cheng, J.; Teply, B. A.; Sherifi, I.; Sung, J.; Luther, G.; Gu, F. X.; Levy-Nissenbaum, E.; Radovic-Moreno, A. F.; Langer, R.; Farokhzad, O. C., Formulation of functionalized PLGA-PEG nanoparticles for in vivo targeted drug delivery. *Biomaterials* **2007,** *28* (5), 869-76.

22. Danhier, F.; Lecouturier, N.; Vroman, B.; Jérôme, C.; Marchand-Brynaert, J.; Feron, O.; Préat, V., Paclitaxel-loaded PEGylated PLGA-based nanoparticles: In vitro and in vivo evaluation. *J. Control. Release* **2009,** *133* (1), 11-17.

23. Buske, J.; König, C.; Bassarab, S.; Lamprecht, A.; Mühlau, S.; Wagner, K. G., Influence of PEG in PEG–PLGA microspheres on particle properties and protein release. *Eur J Pharm Biopharm* **2012,** *81* (1), 57-63.

24. Lehner, E.; Liebau, A.; Menzel, M.; Schmelzer, C. E. H.; Knolle, W.; Scheffler, J.; Binder, W. H.; Plontke, S. K.; Mäder, K., Characterization of PLGA versus PEG-PLGA intracochlear drug delivery implants: Degradation kinetics, morphological changes, and pH alterations. *J Drug Deliv Sci Tec* **2024,** *99*, 105972.

25. Khalil, N. M.; do Nascimento, T. C.; Casa, D. M.; Dalmolin, L. F.; de Mattos, A. C.; Hoss, I.; Romano, M. A.; Mainardes, R. M., Pharmacokinetics of curcumin-loaded PLGA and PLGA-PEG blend nanoparticles after oral administration in rats. *Colloids Surf B Biointerfaces* **2013,** *101*, 353-60.

26. Costello, M. A.; Liu, J.; Chen, B.; Wang, Y.; Qin, B.; Xu, X.; Li, Q.; Lynd, N. A.; Zhang, F., Drug release mechanisms of high-drug-load, melt-extruded dexamethasone intravitreal implants. *Eur J Pharm Biopharm* **2023,** *187*, 46-56.

27. Bhagat, R.; Zhang, J.; Farooq, S.; Li, X. Y., Comparison of the release profile and pharmacokinetics of intact and fragmented dexamethasone intravitreal implants in rabbit eyes. *J Ocul Pharmacol Ther* **2014,** *30* (10), 854-8.

28. Elena de Souza, L.; Eckenstaler, R.; Syrowatka, F.; Beck-Broichsitter, M.; Benndorf, R. A.; Mäder, K., Has PEG-PLGA advantages for the delivery of hydrophobic drugs? Risperidone as an example. *J Drug Deliv Sci Technol* **2021,** *61*.

29. Johnson, C.; Chen, B.; Bhalla, A.; Crowell, E.; Zhang, F., Elimination of "lag" and "burst" phases in drug release profiles of melt-extruded, PLGA-based intravitreal implants. *Int J Pharm* **2025,** *681*, 125822.

30. Bode, C.; Kranz, H.; Kruszka, A.; Siepmann, F.; Siepmann, J., In-situ forming PLGA implants: How additives affect swelling and drug release. *J Drug Deliv Sci Technol* **2019,** *53*, 101180.

31. Desai, K. G. H.; Mallery, S. R.; Schwendeman, S. P., Effect of formulation parameters on 2-methoxyestradiol release from injectable cylindrical poly(dl-lactide-co-glycolide) implants. *Eur J Pharm Biopharm* **2008,** *70* (1), 187-198.

32. Lehner, E.; Honeder, C.; Knolle, W.; Binder, W.; Scheffler, J.; Plontke, S. K.; Liebau, A.; Mader, K., Towards the optimization of drug delivery to the cochlear apex: Influence of polymer and drug selection in biodegradable intracochlear implants. *Int J Pharm* **2023,** *643*, 123268.

33. Lehner, E.; Menzel, M.; Gundel, D.; Plontke, S. K.; Mader, K.; Klehm, J.; Kielstein, H.; Liebau, A., Microimaging of a novel intracochlear drug delivery device in combination with cochlear implants in the human inner ear. *Drug Deliv Transl Res* **2022,** *12* (1), 257-266.

34. Kempe, S.; Mader, K., In situ forming implants - an attractive formulation principle for parenteral depot formulations. *J Control Release* **2012,** *161* (2), 668-79.

35. Sellaturay, P.; Nasser, S.; Islam, S.; Gurugama, P.; Ewan, P. W., Polyethylene glycol (PEG) is a cause of anaphylaxis to the Pfizer/BioNTech mRNA COVID-19 vaccine. *Clin Exp Allergy* **2021,** *51* (6), 861-863.

36. Glassner, M.; Vergaelen, M.; Hoogenboom, R., Poly(2‐oxazoline)s: A comprehensive overview of polymer structures and their physical properties. *Polym Int* **2017,** *67* (1), 32-45.

37. Sedlacek, O.; Monnery, B. D.; Filippov, S. K.; Hoogenboom, R.; Hruby, M., Poly(2-oxazoline)s--are they more advantageous for biomedical applications than other polymers? *Macromol Rapid Commun* **2012,** *33* (19), 1648-62.

38. Hoogenboom, R.; Schlaad, H., Thermoresponsive poly(2-oxazoline)s, polypeptoids, and polypeptides. *Polym Chem-Uk* **2017,** *8* (1), 24-40.

39. Hoogenboom, R., The future of poly(2-oxazoline)s. *Eur Polym J* **2022,** *179*, 111521.

40. Jana, S.; Hoogenboom, R., Poly (2‐oxazoline) s: a comprehensive overview of polymer structures and their physical properties—an update. *Polym Int* **2022,** *71* (8), 935-949.

41. de la Rosa, V. R., Poly(2-oxazoline)s as materials for biomedical applications. *J Mater Sci-Mater M* **2014,** *25* (5), 1211-25.

42. Rettler, E. F. J.; Lambermont-Thijs, H. M. L.; Kranenburg, J. M.; Hoogenboom, R.; Unger, M. V.; Siesler, H. W.; Schubert, U. S., Water uptake of poly(2-N-alkyl-2-oxazoline)s: influence of crystallinity and hydrogen-bonding on the mechanical properties. *J Mater Chem* **2011,** *21* (43), 17331-17337.

43. Kempe, K.; Rettler, E. F. J.; Paulus, R. M.; Kuse, A.; Hoogenboom, R.; Schubert, U. S., A systematic investigation of the effect of side chain branching on the glass transition temperature and mechanical properties of aliphatic (co-)poly(2-oxazoline)s. *Polymer* **2013,** *54* (8), 2036-2042.

44. Hoogenboom, R., Poly(2-oxazoline)s: a polymer class with numerous potential applications. *Angew Chem Int Ed Engl* **2009,** *48* (43), 7978-94.

45. Einzmann, M.; Binder, W. H., Novel functional initiators for oxazoline polymerization. *J Polym Sci Pol Chem* **2001,** *39* (16), 2821-2831.

46. Hoogenboom, R.; Fijten, M. W. M.; Thijs, H. M. L.; Van Lankvelt, B. M.; Schubert, U. S., Microwave-assisted synthesis and properties of a series of poly(2-alkyl-2-oxazoline)s. *Des Monomers Polym* **2005,** *8* (6), 659-671.

47. Jacobsen, S.; Fritz, H.-G., Plasticizing polylactide—the effect of different plasticizers on the mechanical properties. *Polym Eng Sci* **1999,** *39* (7), 1303-1310.

48. Wypych, G., Effect of plasticizers on properties of plasticized materials. In *Handbook of Plasticizers (Fourth Edition)*, Wypych, G., Ed. ChemTec Publishing, Toronto, Canada: 2004; pp 209-332.

49. Ghaffari, A. A.; Matter, B. A.; Hartman, R. R.; Bourne, D. W. A.; Wang, Y.; Choi, S.; Kompella, U. B., Hot-Melt Extrusion-Based Dexamethasone-PLGA Implants: Physicochemical, Physicomechanical, and Surface Morphological Properties and In Vitro Release Corrected for Drug Degradation. *Pharmaceutics* **2024,** *16* (7), 895.

50. Panyam, J.; Williams, D.; Dash, A.; Leslie-Pelecky, D.; Labhasetwar, V., Solid-state solubility influences encapsulation and release of hydrophobic drugs from PLGA/PLA nanoparticles. *J Pharm Sci* **2004,** *93* (7), 1804-14.

51. Liang, D.; Frank, S.; Schwendeman, S. P., Aqueous remote loading of model cationic peptides in uncapped poly(lactide-co-glycolide) microspheres for long-term controlled release. *Drug Deliv Transl Res* **2024,** *14* (3), 696-704.

52. Lehner, E.; Liebau, A.; Syrowatka, F.; Knolle, W.; Plontke, S. K.; Mader, K., Novel biodegradable Round Window Disks for inner ear delivery of dexamethasone. *Int J Pharm* **2021,** *594*, 120180.
